# An Overview of Ischemia–Reperfusion Injury: Review on Oxidative Stress and Inflammatory Response

**DOI:** 10.5152/eurasianjmed.2022.22293

**Published:** 2022-12-01

**Authors:** Mustafa Can Güler, Ayhan Tanyeli, Fazile Nur Ekinci Akdemir, Ersen Eraslan, Saime Özbek Şebin, Derya Güzel Erdoğan, Tuncer Nacar

**Affiliations:** 1 Department of Physiology, Atatürk University Faculty of Medicine, Erzurum, Turkey; 2 Department of Nutrition and Dietetics, Ağrı İbrahim Çeçen University Faculty of Medicine, Ağrı, Turkey; 3 Department of Physiology, Yozgat Bozok University Faculty of Medicine, Yozgat, Turkey; 4 Department of Physiology, Sakarya University Faculty of Medicine, Sakarya, Turkey; 5 Department of Physiology, Yüksek İhtisas University Faculty of Medicine, Ankara, Turkey

**Keywords:** Inflammatory response, ischemia–reperfusion injury, oxidative stress, reactive oxygen species

## Abstract

Ischemia–reperfusion is a common health problem leading to several health conditions. The pathophysiology of ischemia–reperfusion is quite complex. Oxidative stress and inflammatory response contribute to ischemia–reperfusion mechanisms. Various parameters like proinflammatory cytokines, reactive oxygen species, occur during ischemia–reperfusion . There are several ways to investigate these values through biochemical and histopathologic findings. Malondialdehyde, glutathione, myeloperoxidase, superoxide dismutase, interleukin 6, interleukin 1β, tumor necrosis factor alpha, caspase-3, nuclear factor-kappa β, and LC3B (microtubule-associated protein light chain 3, LC3) can be evaluated among these indicators.

**Main Points**

- Ischemia–reperfusion (I/R) is a severe condition with a wide range of clinical tables. Although various health problems originate from I/R, examining common parameters would help to understand the nature of I/R.
- Ischemia–reperfusion injury involves oxidative stress-related results and inflammatory response. Examining the related parameters in different types of I/R injuries may allow comparison and understanding.
- Moreover, considering oxidative stress and inflammatory response also helps link up these processes rather than evaluating them as separate events.

## Introduction

Early in the 19th century, the term “ischemia” was first described as inadequate blood supply to tissues caused by obstruction of the arterial inflow.^1^ Ischemia occurs when blood flow in tissues or organs decreases or stops completely.^2^ All tissues can withstand brief periods of ischemia. Cell injury and/or death occur after a critical period of ischemia, which varies depending on the cell type and organ.^3^

Reperfusion, which restores oxygen and nutrients to cells and removes metabolic by-products, can cause pathogenetic processes that worsen ischemia damage.^3^ Reperfusion exacerbates ischemic damage by increasing oxidative stress and inflammation.^4^ It may damage distant organs due to mediator release from vascularized tissues into the blood.^3^ Reperfusion injury is complicated and includes inflammatory response and reactive oxygen species (ROS) formation due to reoxygenation.^5^ The reperfusion is a dynamic process with cell death lasting up to 3 days after it starts.^6^

Ischemia–reperfusion (I/R) damage causes cell dysfunction, tissue damage, cell death, and organ dysfunction by preventing ischemia-affected cells from receiving oxygen and nutrients due to impaired blood flow.^7,8^ I/R injury is a multifactorial inflammatory process with high mortality and morbidity rates and can cause acute organ dysfunction.^9,10^ Various conditions and procedures, including organ transplantation, low cardiac output, and shock, can cause I/R.^11^ Experimental I/R models show that injury response after reperfusion correlates with the ischemia period.^12^

I/R damage occurs in 2 modes. If the adaptive threshold of the cell to utilize anaerobic metabolism is exceeded in the initial ischemic phase, it causes cellular dysfunction and irreversible damage or necrosis. In the reperfusion phase following ischemia, the ischemic injury is overcome by reinstating blood flow to prevent viable ischemic tissue. As it is, ROS generation can exacerbate the damage due to an intense immune response and inflammation.^1,13^

## Reactive Oxygen Species and Oxidative Stress in Ischemia–Reperfusion Injury

ROS are reactive and potentially dangerous oxidant molecules such as superoxide, hydroxyl radical, singlet oxygen, and hydrogen peroxide in living cells.^14,15^ They participate in reactions related to production and destruction in the body.^16^ ROS are a crucial regulator of cellular signaling and energy delivery in physiological conditions.^14,17^ ROS play an essential role in the pathophysiology of I/R injury. I/R-induced ROS injury interacts with various lipids and proteins to generate oxidative stress.^18^

Oxidative stress is an unbalance of the oxidant system that accelerates the reaction of ROS with cellular macromolecules, resulting in the disruption of oxidant–antioxidant homeostasis. It occurs when there is an overproduction of oxidants or a decline in antioxidants.^14,19,20^ Peroxidation is one of the most essential and typical consequences of oxidative stress.^21^ Malondialdehyde (MDA) is the most mutagenic of the aldehydes that can be produced as secondary products during lipid peroxidation.^22^ MDA is a marker for oxidative stress and protracted cell damage.^23,24^

Myeloperoxidase (MPO) is a heme peroxidase enzyme that is found in neutrophils and monocytes' azurophilic granules in large amounts.^25^ It is a crucial component of the phagocytic microorganism-killing efficacy of the innate immune system.^26^ Polymorphonuclear neutrophil infiltration is characteristic of acute injury caused by tissue I/R, drug toxicity, shock, and similar causes.^27^ In acute inﬂammatory conditions, MPO is released in the extracellular medium.^28^ MPO levels rise as a result of neutrophil migration and activation by I/R.^29^ Several studies have reported I/R related high MPO measurements.^30-33^

Excess ROS and superoxide can upset the balance and eventually induce oxidative stress. Superoxide is the beginning of a series of free radical processes resulting in ROS's uncontrolled production. Superoxide dismutase (SOD) is the initial antioxidant defense to act against superoxide and subsequent oxidative stress.^34^

Glutathione (GSH) is an endogenous peptide with antioxidant and other metabolic functions.^35^ Glutathione’s potent antioxidant properties protect the cell, particularly the cell membrane, from free radical damage. Glutathione is an antioxidant but also participates in the immune response and in repairing and protecting DNA.^34^ Glutathione levels diminish with aging, associated with increased oxidative damage.^36^ Experimental I/R models represented significant reductions in GSH value, SOD activity, and other antioxidant levels.^37,38^

## Inflammatory Process and Immune Response

I/R induces inflammation. Inflammation is necessary for the defense against invading pathogens. Neutrophils and macrophages phagocytose the source of infection, lymphocytes are activated, adaptive immune responses occur, and additional cytokines and chemokines are produced as a result of a cascade of signals that occurs in response to an infection.^39^ The inflammatory response is necessary for wound and tissue repair. It typically occurs without microorganisms and is therefore referred to as sterile inflammation. I/R-related sterile inflammation includes significant neutrophil formation, cytokine production, and other proinflammatory stimuli.^39^

Tumor necrosis factor-alpha (TNF-α) and interleukin-1 (IL-1) are proinflammatory cytokines attending acute inflammation.^40^ They are part of large networks that positively and negatively affect the cells. They increase endothelial permeability and facilitate leukocyte infiltration.^40^

TNF-α was first identified as lymphocyte and macrophage product that can lyse certain cell types, particularly tumor cells.^41^ It is linked to a wide variety of physiological processes present in healthy and diseased bodies.^42,43^ TNF-α is an essential cell signaling molecule with various roles in several tissues. It is involved in the induction of systemic inflammation and apoptosis.^44,45^ TNF-α is upregulated in response to ischemic injury. It controls fundamental biological functions such as apoptosis, cell proliferation, immune response, and differentiation. It is strongly linked to the occurrence of oxidative stress.^46^

Interleukin-1 beta (IL-1β) is initially identified as an endogenous pyrogen-inducing fever in rabbits.^47^ Almost all nucleated cells produce IL-1β.^48^ IL-1β is released into the microenvironment by immune system cells to provide paracrine or autocrine regulation in response to inflammation.^49^ IL-1β transcription can be enhanced by proinflammatory stimuli and proinflammatory cytokines such as type I interferons and TNF-α.^50-52^ IL-1β is produced during infection, injury, or as an immunological response. It causes hypotension, fever, and the production of various proinflammatory cytokines, such as IL-6, at minimal concentrations.^53,54^ TNF-α and IL-1β elevate in the onset of inflammation.^55^

IL-6 is a pleiotropic cytokine with multiple functions. It is produced by monocytes, activated B and T lymphocytes, fibroblasts, and activated macrophages.^56^ Interleukin-6 is produced during infections and tissue injury.^57^ It affects the immune system, inflammation, and hematopoiesis in a variety of ways.^58^ IL-1β, TNF-α, and IL-6 levels were found to be higher in various I/R studies.^59-63^

Nuclear factor-kappa B (NF-κB) was first discovered as a linear transcription factor in B cells of lymphocytes connecting to the light chain enhancer of the kappa immunoglobulin gene. Numerous processes, including inflammation, protection against apoptosis, and the host's immune response, are regulated by NF-κB. Disruptions in NF-κB signaling bring diseases of the immune system, inflammation, and infection.^64^ Interleukin-1 and TNF receptors can activate NF-κB signaling, which is required for inflammatory mechanisms and a key factor in controlling innate immunity.^65^ On the other side, NF-κB promotes IL-1, IL-6, and TNF-α expression.^66,67^ Numerous experimental models investigated NF-κB levels in terms of inflammation, infection, and immune system evaluation.^68,69^

## Cell Death Pathways in Ischemia–Reperfusion Injury

Extrinsic factors like energy depletion, inflammatory mediators and toxic molecule production, and mechanical injury were thought to cause I/R-induced cell death for years.^1^ Cells can also be scheduled to die by cellular signaling pathways via processes such as autophagy and apoptosis.^70^

Tissue development and homeostasis depend heavily on apoptosis. Apoptosis has the purpose of ridding an organism of harmful cells, such as virus-infected and genetically altered cells.^71^ Reperfusion degrades mitochondria due to excessive ROS and reduced ATP production. Lipid damage and oxidative stress damage mitochondrial DNA, membrane permeability, and cell death through apoptosis.^40^ Caspase-3 is known to correlate well with apoptosis.^71^

Caspase-3 is a protein encoded by the CASP3 gene.^72^ Caspase-3, a member of the cysteine protease family, has been identified as a critical effector enzyme in the induction of cell apoptosis. Caspase-3 is present in viable cells as an inactive pro-caspase activated during apoptosis, resulting in cell death.^73^ It is activated by caspases 8, 9, and 10 in response to apoptotic signaling events.^74^

Autophagy is crucial for cells and organisms to perform homeostatic tasks and deal with stress.^75^ Autophagy is cells' primary “housekeeping” mechanism, removing dysfunctional organelles and protein aggregates.^1^ Autophagy involves transporting intracellular components to the lysosome for recycling and degradation. Autophagy-related proteins carry out the autophagic program.^75^

Although generally thought of as a critical mechanism for cellular survival, autophagy may also play a direct role in cellular death in certain situations (autophagic cell death).^76^ Autophagy that is not correctly regulated will, in the end, result in the cell's death and may also contribute to I/R injury.^1^ Three isoforms of microtubule-associated protein light chain 3 (MAPLC3, LC3) exist (LC3A, LC3B, and LC3C). One of the most popular indicators of autophagy is LC3B.^77^ Due to its wide tissue specificity and distinct localization on autophagosomes, LC3B is frequently used as an autophagosome marker.^78^ Numerous animal I/R models in the scientific literature exhibited elevated caspase-3 and LC3B.^32,33,60^

## Conclusion

I/R injury is a process that can occur simultaneously or sequentially in several organs in the body. Various parameters detect changes in this process, including oxidative stress, inflammatory response, apoptosis, and autophagy. These investigated parameters provide essential information regarding the formation of I/R, its mechanism of action, and the damage that it causes.

### Search strategy and selection criteria

We searched PubMed and ScienceDirect for literature published between 1987 and 2022 that focused on I/R injury, mostly current articles.
